# Structure factors of random hard disk packing in 2D by explicit modeling

**DOI:** 10.1107/S1600576725002407

**Published:** 2025-04-15

**Authors:** Yoshiharu Nishiyama

**Affiliations:** a Univ. Grenoble Alpes, CNRS, CERMAV, 38000Grenoble, France; Argonne National Laboratory, USA

**Keywords:** structure factors, random packing, parallel cylinders, X-ray/neutron scattering intensity simulation

## Abstract

This paper describes a method that can (1) generate random packing of hard disks in 2D using Monte Carlo simulation, (2) extract the corresponding pair distribution function using normalization by disk line picking probability and (3) convert it to the structure factor. The generated structure factor agrees well with the analytical form based on the Percus–Yevick equation at a low area fraction (that is, within 1% at an area fraction below 0.2 and 2% at an area fraction of 0.3) but differs at a higher area fraction with more pronounced peaks and oscillations. Above an area fraction of 0.69, the hexagonal packing feature appears as sharp peaks at low *Q*, which are absent in the analytical solution. The structure factors up to an area fraction of 0.65 as a function of *QR* and the area fraction are stored in table form. The structure factor table can be combined with the cylinder form factors to simulate the X-ray/neutron scattering intensity of wood cell wall scattering.

## Abstract 2

Structure factors of disks that are randomly packed in 2D, equivalent to parallel cylinders, are given as a function of the area fraction. This structure factor can be used to interpret scattering features from densely packed fibrous systems.

## 1. Introduction

Determining the shape/dimension of the particles and their arrangement in a dense system of particles by small-angle scattering techniques is challenging since the characteristic features of form factors and structure factors appear on the same length scale, sometimes leading to misinterpretation of X-ray/neutron scattering data. An example is the packing of cellulose microfibrils in wood cell walls with a volume fraction of typically 30–50% (Rowell *et al.*, 2012 ▸). Ultimately, there is no unique solution to simultaneously determine the particle shape and structure factor if neither is known.

The arrangement of hard disks, the equivalent of hard spheres in 2D, is what Metropolis studied in his seminal work on the Monte Carlo algorithm in 1953 (Metropolis *et al.*, 1953 ▸). Since Monte Carlo simulation that involved explicitly calculating the arrangement of particles was expensive at the time, more analytical approaches using integral equations were also developed (Lado, 1968 ▸). The Percus–Yevick (PY) approximation is one such method that leads to an analytical pair distribution function (PDF) which shows relatively good agreement with the Monte Carlo simulation, except for the underestimation of the population at contact (Lado, 1968 ▸).

Rosenfeld (1990 ▸) proposed an even simpler analytical form that reproduces the structure factor of the PY approximation at low number density but fails at higher density. Here the structure factor 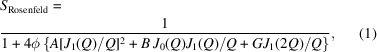
where ϕ is the area fraction, *J_n_* is the *n*th-order Bessel function and 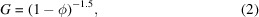

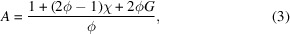

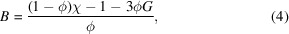

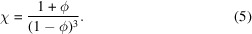


Another possibility is to start with hexagonal packing and introduce an analytical form of disorder of the second kind (*i.e.* paracrystallinity) as proposed by Hashimoto *et al.* (1994 ▸). Penttilä *et al.* (2019 ▸) incorporated this in the WoodSAS model to fit X-ray scattering from wood. This allows the extraction of parameters such as lattice constants and disorder parameters. This paracrystalline model is adapted for a crystallizing system with well defined crystalline peaks that broaden at higher resolution. However, Penttilä *et al.* (2019 ▸) had to artificially trim the central scattering that tends to significantly deviate toward high intensity at low *Q* using the original mathematical model.

Since the structure factor of a hard disk liquid is a smooth function of the scattering vector magnitude *Q*, one can also establish a sparse tabulation to accurately reproduce the structure factor by a spline function. Here, we report a method to explore the liquid structure factor via Monte Carlo simulation and store the data in a readily utilizable form, which can be a more physical model than the ‘paracrystalline hexagonal packing’ in many cases. This type of packing is typical of fibrillar systems without a strong long-range interaction, for example sterically stabilized colloidal systems (Grelet & Rana, 2016 ▸) or biological systems such as collagen fibril packing (Meek & Boote, 2009 ▸).

## 2. Methods

### 2.1. Generation of random distribution

The Monte Carlo simulation performed here is very similar to what was originally used by Metropolis, with small modifications. The disks are initially positioned on a hexagonal array within a square periodic boundary box, and they are then moved in random steps under the condition of not colliding with another particle. In the following, we take the radius of the disk as a unit of length. In the case of a radius that is not one unit long, *Q* can be replaced by *QR* where *R* is the radius. Given a target volume (area) fraction ϕ_0_, the center-to-center distance, *a*, between disks in a hexagonal packing is 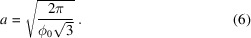
We can fill a square with side length *l*, chosen as *l* = 500 here, with 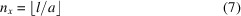
disks in the horizontal direction and 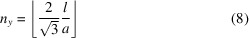
discs in the vertical direction. This corresponds to a real volume fraction 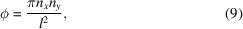
which is slightly smaller than ϕ_0_. The step size *v_i_* along the *x* and *y* directions follows a normal probability function: 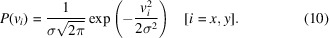
At each step, one randomly chosen disk is moved by a step (*v_x_, *v_y_**) and we check for a collision with other disks. If the distance between two disks is smaller than two, this move is rejected and the disk returns to the initial position. σ is taken as *a*/2 − 1 but is reduced if more than 50% of the movements are rejected: 10*N* moves were applied here, where *N* = *n_x_*n_y_** is the number of disks, to check the rejection ratio. When the rejection rate was greater than 50%, σ was multiplied by 0.8 and the rejection rate was checked again. This operation was repeated until the rejection rate was below 50%.

#### 2.1.1. Collision check

To minimize the number of distance calculations for collision check, the periodic boundary is divided into tiles with side length 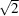, which can accommodate only one disk. A unique serial number is assigned to each disk whose current coordinates are stored in memory. In total (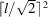) tiles are represented in an integer array that hosts −1 if there is no disk at the position and the serial number of the disk if there is one. To check the collision, we only need to verify 20 tiles whose center-to-center distance is within a distance of 4, excluding the tile on which the disk that is moved sits, and calculate the distance only if there is a disk on the tile.

### 2.2. Pair distribution function

A histogram of the distances between particles for all pairs of particles is calculated within a circle centered on the periodic boundary box with a diameter of *l*. This corresponds to 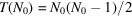 distance calculations, where *N*_0_ is the number of particles in the circle. This histogram was normalized by 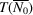 using a theoretical number 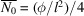 instead of *N*_0_, which fluctuates around the value 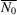. The width of the histogram bin *w*_b_ was 0.1, with the total number of bins *N*_b_ = 1/*w*_b_. The normalized histogram is an array of probabilities *p_i_* for each bin *i*, which sum to 1:
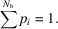


The probability density of the distance between two random points, ρ(*r*), in a circle with radius *R*, is (Mathai, 1999 ▸) 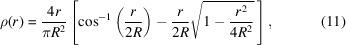
which can be further simplified in discrete form with 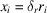 as 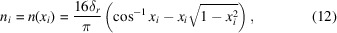
where *n_i_* is the expected count in the *i*th bin and δ_*r*_ is the bin width divided by 2*R*, or 1/*N*_b_.

Finally, the discrete PDF is obtained by dividing each histogram element by *n*(*x_i_*): 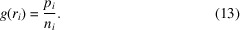
*n_i_* only needs to be calculated once, and thus the calculation time is essentially spent obtaining the histogram. The discrete PDF was calculated and stored every 50*N* moves.

### 2.3. Structure factor calculation

The structure factor *S* of a two-dimensional isotropic or cylindrically symmetric system in the radial direction is (Oster & Riley, 1952 ▸) 
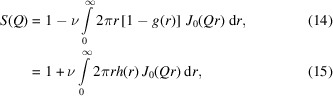
where *g*(*r*) is the PDF and *h*(*r*) = *g*(*r*) − 1 is the correlation function, *J*_0_ is the Bessel function of zero order, and ν is the number density of the disks. *g*(*r*) tends to 1 at large distance *r*, and thus *h*(*r*) tends toward 0. Thus, integration can be stopped at some finite distance *r*. In the discrete form, 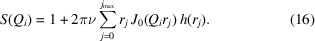
*r_j_J*_0_(*Q_i_r_j_*) can be stored in a matrix 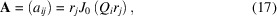
such that the structure factor array **S** can be obtained by simple matrix operation: 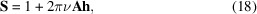
where the *i*th element of **h** is *h*(*r*_*j*_) of equation (16). The extent of *r*, *r*_max_, was typically chosen to be *l*/2. *g*(*r*) was linearly scaled down from *l*/4 to reach 0 at *l*/2 to reduce noise at low *Q*, but was also varied between 5 and 200 to check the influence on the calculated structure factor.

### 2.4. Spline fitting

Due to the oscillation in the very low *Q* region, the numerically calculated structure factors below *Q* = 0.6 were fitted to a parabolic function 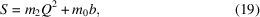
with the weighting proportional to *Q*^2^, and the direct results from the simulations were replaced with the values according to (19). The one-dimensional curves were smoothed with a Savitzky–Golay filter (Savitzky & Golay, 1964 ▸) using a window width of 29 and using the Python package *savgol_filter* from the *SciPy* library (Virtanen *et al.*, 2020 ▸) to decrease the numerical noise. Curves over the whole *Q* range from 0 to 10 and for an area fraction from 0.05 to 0.65 were approximated with a bivariate smoothing second-order B spline using the *bisplrep* function from *SciPy*. The weight was set to unity for all points with smoothing factor 0.1.

## 3. Results

### 3.1. Pair correlation function

**Figure 1:**
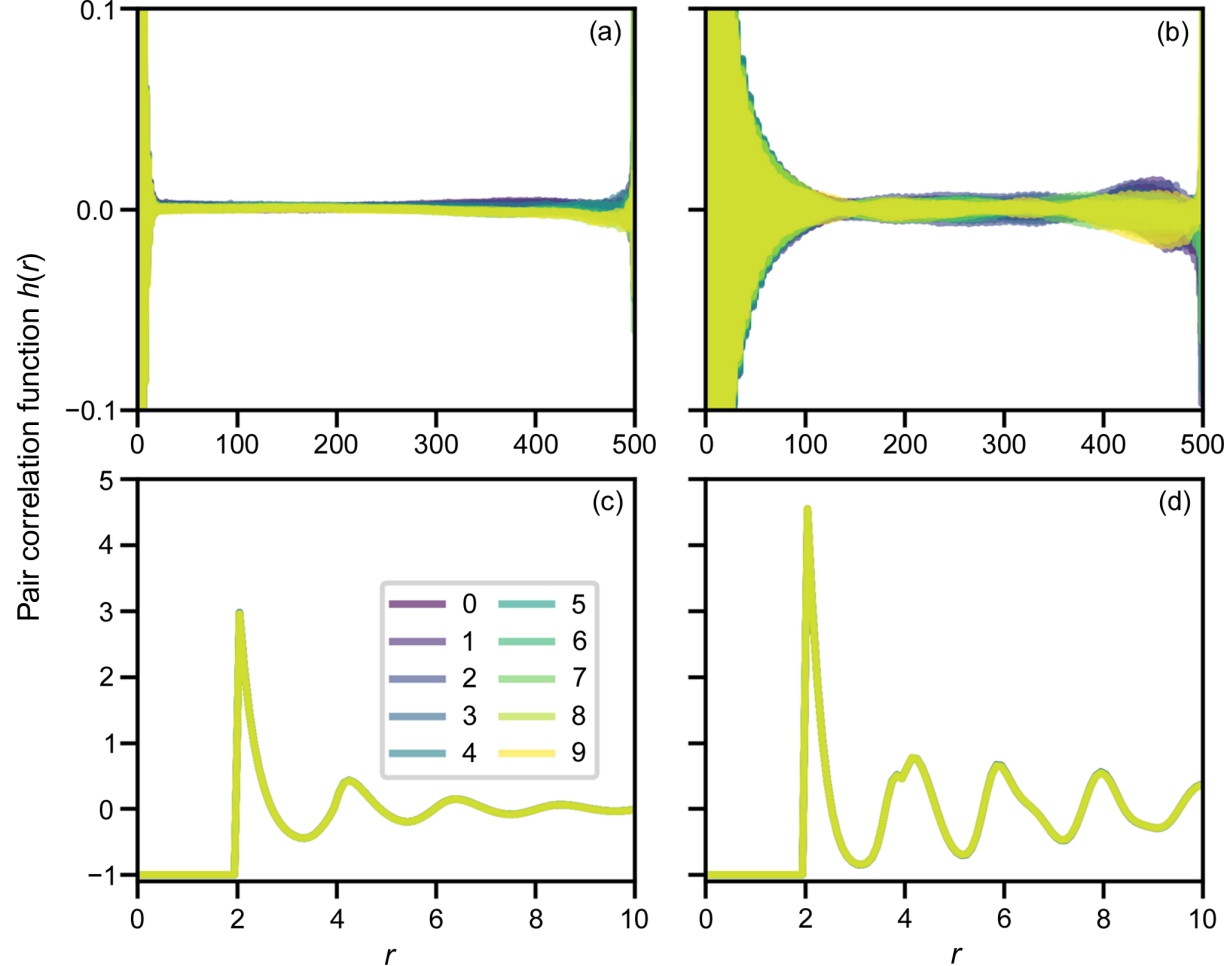
Pair correlation function of random disks with the area fractions (*a*) 0.6 and (*b*) 0.71. Each curve is an average of 100 consecutive pair correlation functions; (*c*) and (*d*) are enlargements of the small-distance regions of (*a*) and (*b*), respectively. The 10 lines correspond to 10 segments of the run.

Fig. 1 ▸ shows the averages of 100 consecutive pair correlation functions calculated for an area fraction of 0.6 [Fig. 1 ▸(*a*)] and 0.71 [Fig. 1 ▸(*b*)], sequentially selected from 1000 data points. There is an almost perfect superposition up to *r* = 250 but, at larger distances, slow fluctuation can be seen in addition to the white noise close to 500 because of the small sampling number. Also, due to the periodic boundary conditions, disks at a distance well beyond half the box size correlate as they are at a shorter distance on the other side of the boundary. Thus, a small oscillation can be seen close to the maximum distance limit.

The corresponding short-distance part of the pair correlation function is enlarged in Figs. 1 ▸(*c*) and 1 ▸(*d*), in which all curves exactly superpose for a given system.

### 3.2. Influence of integration limit on the structure factors

**Figure 2:**
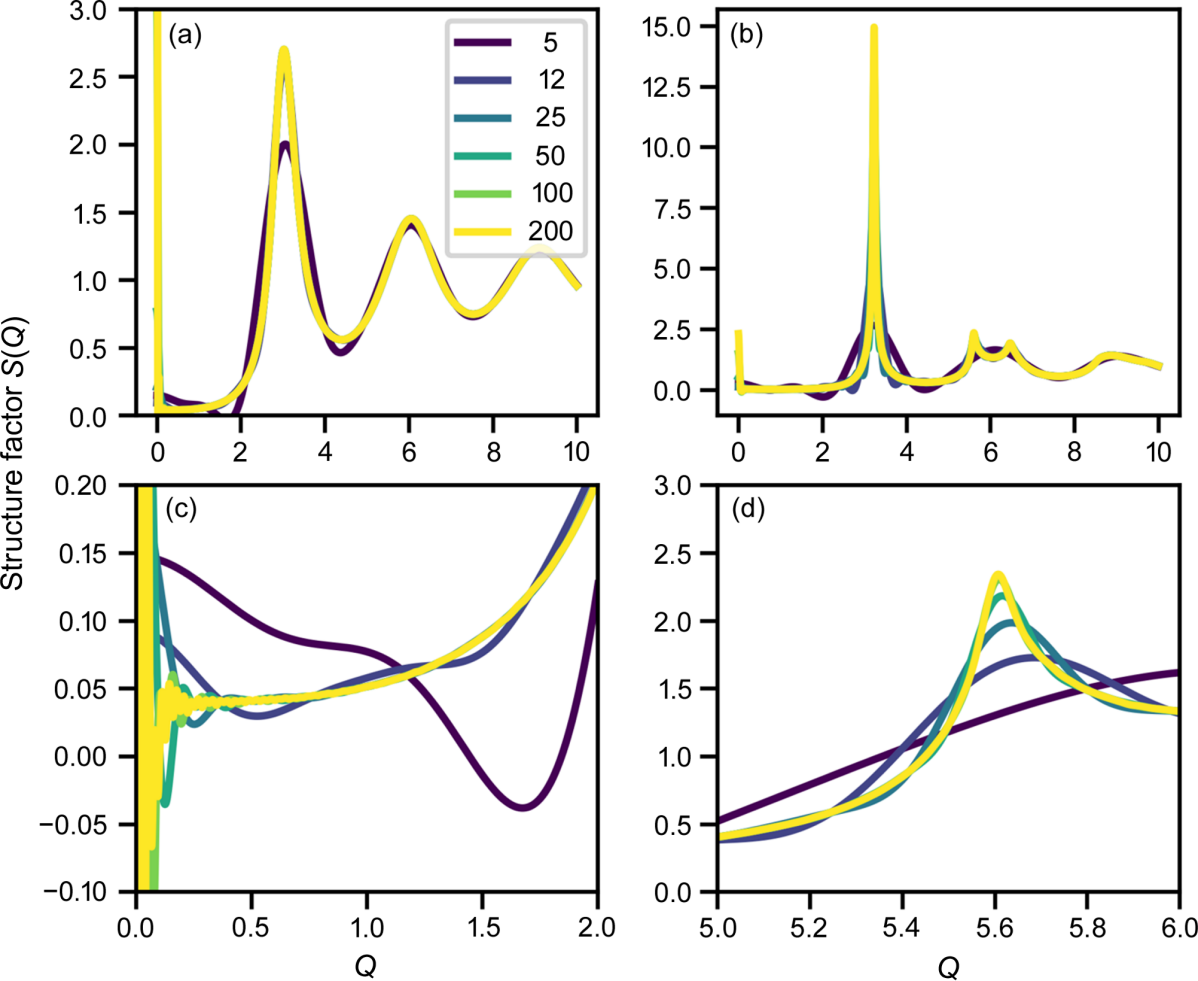
Structure factors corresponding to Fig. 1 ▸, with the area fractions (*a*) 0.6 and (*b*) 0.71, calculated with different integration limits in *r*, from 5 to 200, distinguished by color; (*c*) and (*d*) are zoomed-in images of (*a*) and (*b*), respectively. *Q* = *QR* as *R* = 1.

Fig. 2 ▸ shows the average structure factors corresponding to the pair distributions in Fig. 1 ▸ calculated according to equation (18) using the different extents of *r*, equivalent to truncating the right side of the matrix **A** and the bottom of the vector **h**. Structure factors were calculated for 1000 PDFs and then averaged. At an area fraction of 0.6 [Figs. 2 ▸(*a*) and 2 ▸(*c*)], there is almost no difference beyond the integration limit of 25, except at very small *Q* where the oscillation started at a smaller *Q* with a larger integration limit. However, at higher density with an area fraction of 0.71, taking a smaller limit than 100 starts to smear out the peak around *Q* = 5.6 [Fig. 2 ▸(*d*)] and also introduces oscillation around the main peak.

### 3.3. Structure factors as a function of area fraction

**Figure 3:**
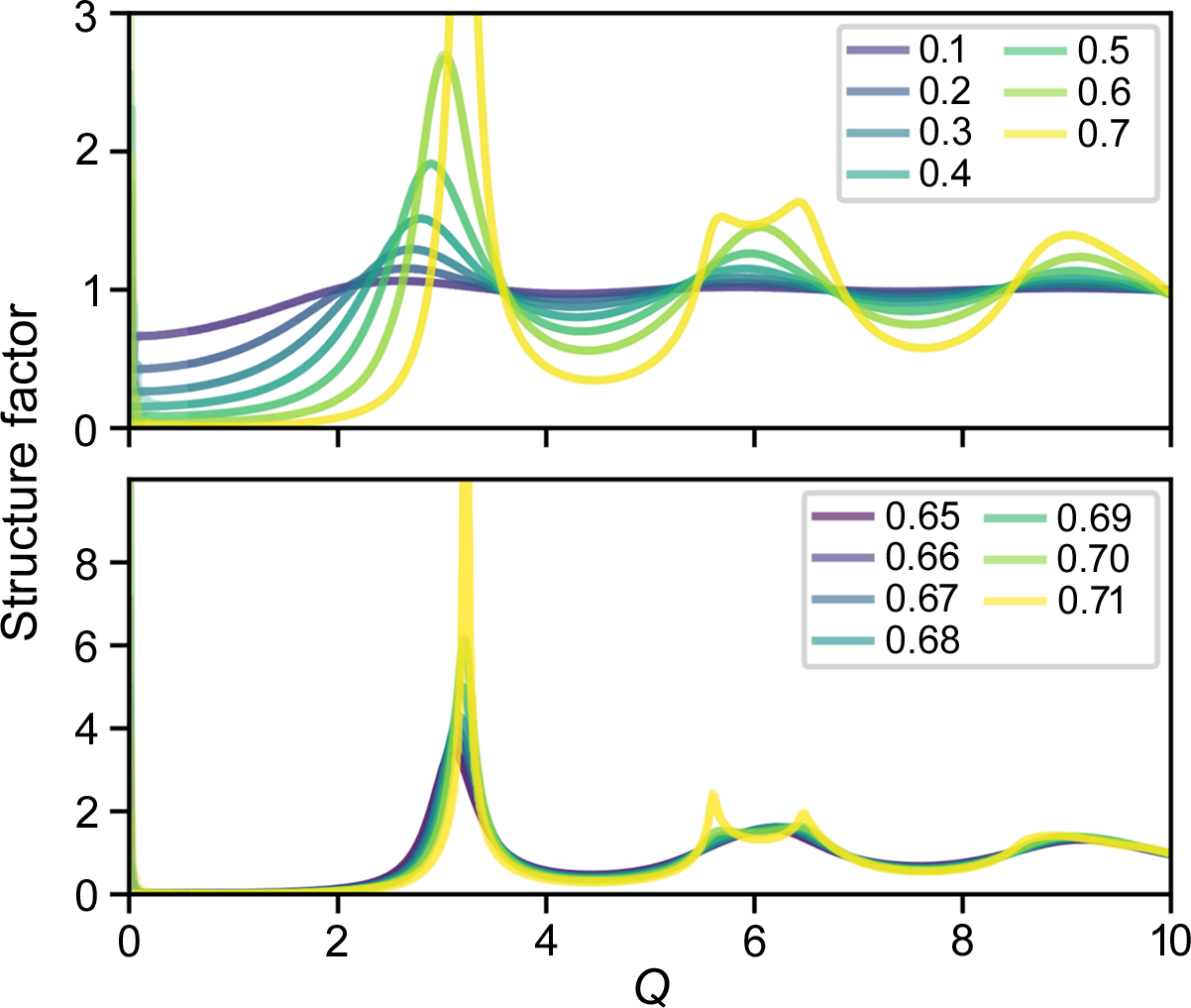
Structure factors of random disks at different area fractions. Spline fitting curves are superposed on the simulation data as faint solid lines (visible only at low *Q*).

Fig. 3 ▸ shows the structure factors of the disks as a function of area fraction, which exhibit more and more pronounced features with increasing area fraction. At an area fraction of 0.71, three sharp peaks at *Q* = 3.24, 5.61 and 6.47, which correspond to the typical *Q* ratios of 1, 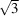 and 2 of a hexagonal lattice, are present. The diffraction features fade away at higher *Q*.

## 4. Discussion

### 4.1. Comparison with the previous structure factor calculations

The PDF and the structure factor at an area fraction of about 0.6 agree well with that reported by Lado (1968 ▸) using a 192-molecule constant-pressure system. The structure factor based on this calculation reported by Rosenfeld (1990 ▸) at ϕ = 0.6 also agrees with our calculation of the structure factor. Using a larger system than Lado (1968 ▸) is unnecessary for this area fraction because the pair correlation function fades out quickly, as seen in Fig. 1 ▸.

Above ϕ = 0.69, a larger system size was necessary to capture the structure since the pair correlation function extends over a long distance, giving rise to a few sharp peaks corresponding to the hexagonal lattice. In addition, the structure evolves slowly at this level of crowding and might be jammed into a state dependent on the sample’s history. A more disordered state might be achievable by other methods, such as the expansion of disks from a given configuration to achieve a high area fraction (Weber *et al.*, 1995 ▸; Torquato *et al.*, 2000 ▸), which is not addressed in this work.

**Figure 4:**
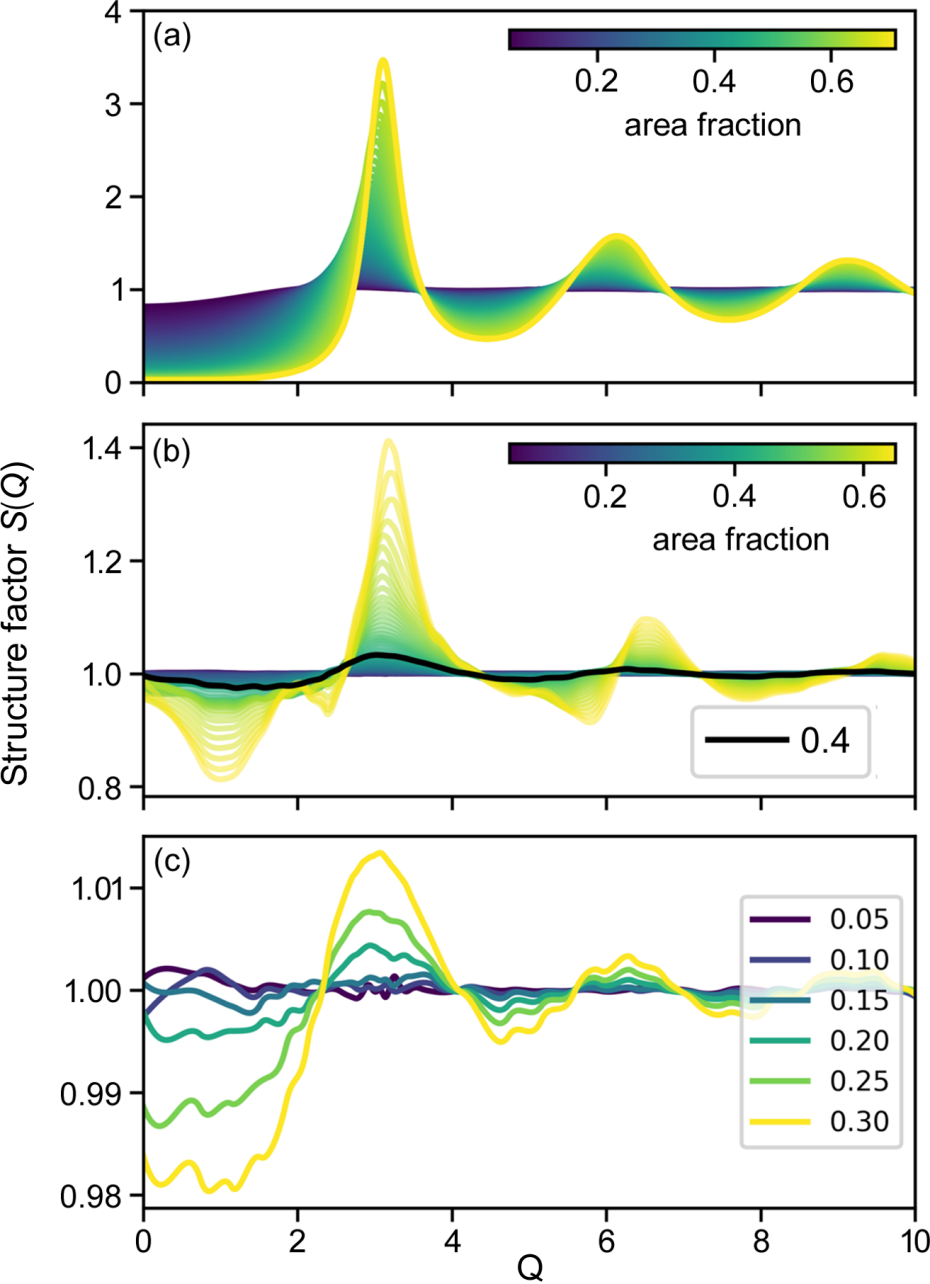
Smoothed and interpolated curves of structure factors as a function of area fraction from 0.05 to 0.7. (*a*) Interpolated structure factors. (*b*) Relative difference from the analytical function provided by Rosenfeld (1990 ▸) in the ϕ range 0.05 to 0.65. (*c*) Same as (*b*) but for ϕ below 0.3.

Fig. 4 ▸(*a*) shows a general view of the interpolated structure factors within the simulated range. The tabulated structure factor data are provided in binary form to be directly loaded as a *numpy* array. Fig. 4 ▸(*b*) shows the ratio of the interpolated structure factor over *S*_Rosenfeld_ of equation (1). The structure factor ratio at 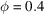, marked in black, shows that the relative difference from the analytical form is within 5% at this area fraction. At smaller ϕ, the agreement is even better: below 2% when ϕ < 0.3 and below 1% when ϕ < 0.2. The deviation becomes more and more pronounced at higher disk densities.

**Figure 5:**
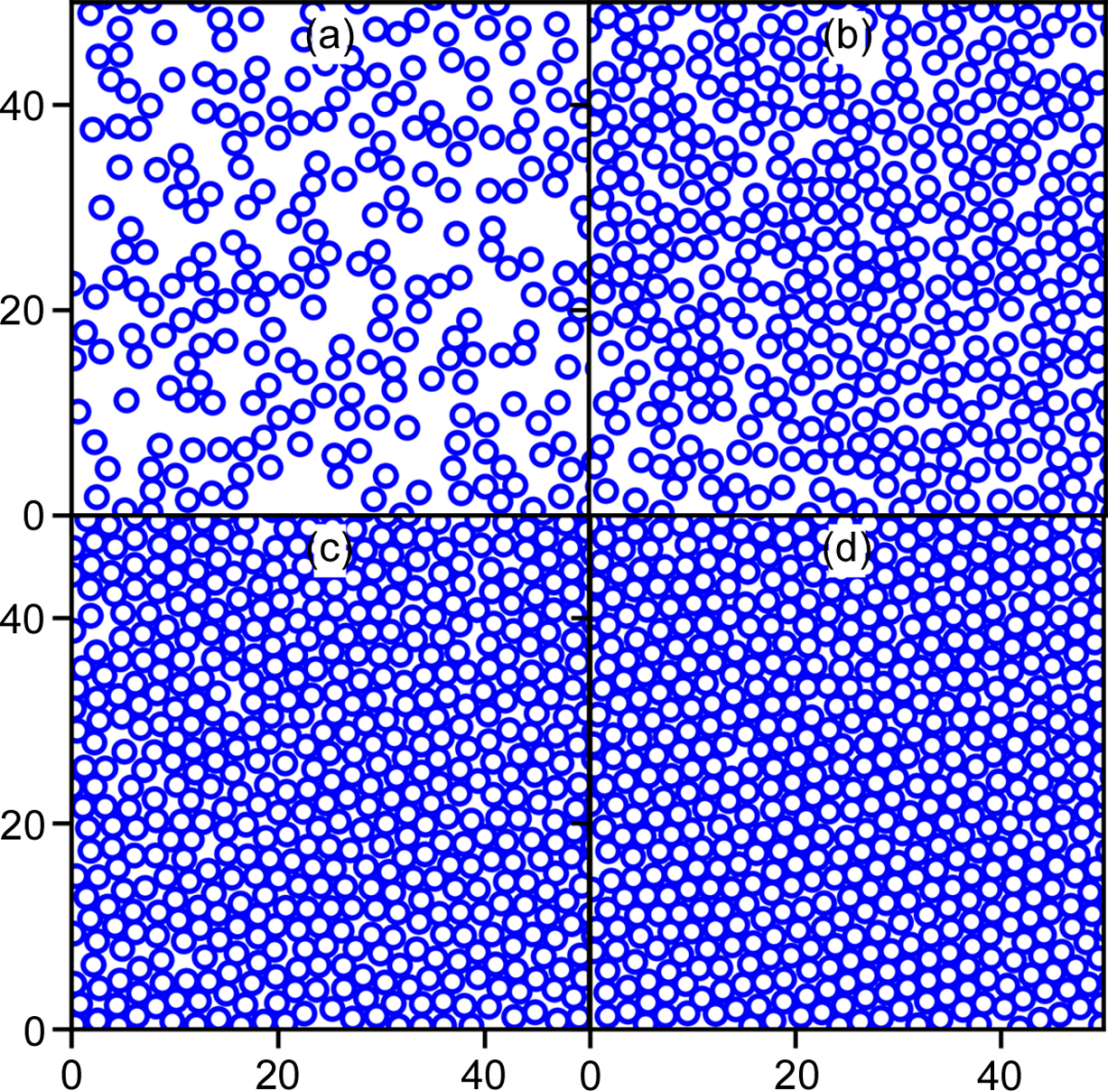
Snapshots of randomly distributed disks at the area fractions (*a*) 0.35, (*b*) 0.5, (*c*) 0.65 and (*d*) 0.7.

One of the advantages of explicit simulation is visualization in real space. Fig. 5 ▸ shows snapshots of the disk dispositions at different disk densities. It can be seen that up to an area fraction of 0.5 [Figs. 5 ▸(*a*) and 5 ▸(*b*)] there is not much occurrence of hexagonal arrangements. At higher densities [Figs. 5 ▸(*c*) and 5 ▸(*d*)], the figures show some regularity and impression of the ‘crystalline’ zone, but even at such regular filling, the structure factor fades away quickly as seen in Figs. 3 ▸ and 4 ▸(*a*).

### 4.2. Interpretation of X-ray correlation peak

**Figure 6:**
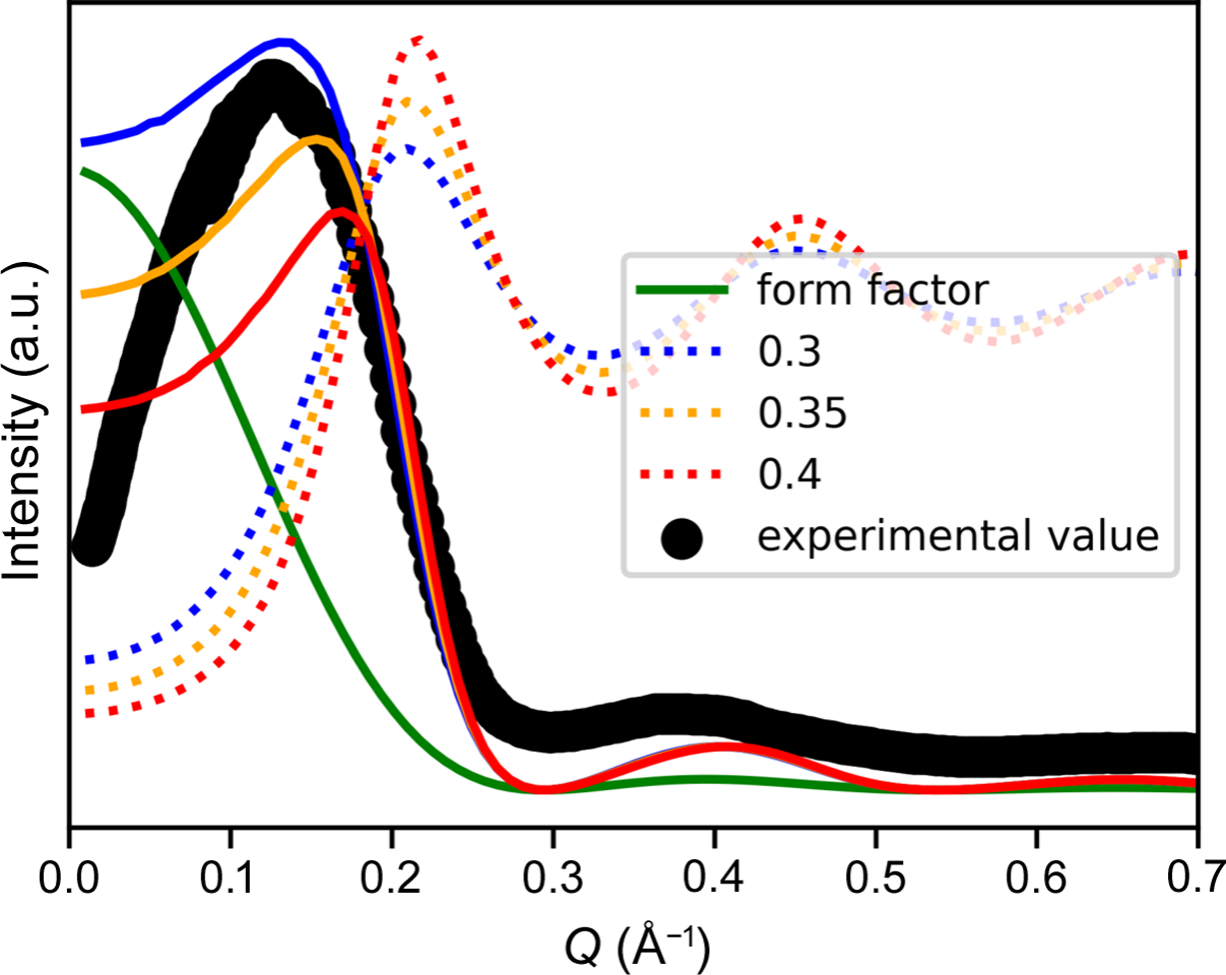
Comparison of X-ray small-angle scattering data of birch wood reported by Chen *et al.* (2021 ▸) with calculated structure factors and the form factor assuming a random packing of infinitely long cylinders with a radius of 13 Å. The intensity is multiplied by *Q* to compensate for the measured intensity being an average over an azimuthal angle, while the model assumes a line trace of a perfectly aligned system.

Fig. 6 ▸ shows the experimental X-ray scattering of native birch wood. When the anisotropic component is isolated and the orientation correction applied, there is a clear correlation peak at *Q* = 0.15. This peak is often interpreted as representing the interfibrillar distances. However, this peak can also be reproduced by multiplying the structure factor with the cylinder form factor 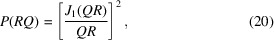
drawn in green in the figure arbitrarily assuming *R* = 1.3 nm. The dotted profiles show the structure factors calculated above, replacing *Q* with *QR* for different area fractions. The expected intensity profiles
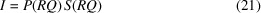
are shown as solid lines. The peak position of the scattering does not correspond to the peak of the structure factor, which is about 0.21 Å^−1^ (2.75 on the *QR* scale) in this area fraction range. However, the peak of the product of the structure factor and form factor is much closer to that of the experimental scattering data, and the position shifts to lower *Q* for a lower volume fraction. The same type of small-angle peak is reported (Kuribayashi *et al.*, 2023 ▸) in a wide range of wood samples with some variation in peak positions, which can also be changed by hydrothermal treatment.

## 5. Conclusions

An exhaustive simulation of the random packing of hard disks in a plane confirmed the validity of Rosenfeld’s analytical expression of the structure factor at small area fractions ϕ < 0.3. However, the analytical form deviates at higher volume fractions. The interpolation of structure factors calculated for a limited number of models provides an alternative to the analytical form and allows further interpretation of scattering from dense systems where the form factor and structure factor interfere. This approach can be further extended for softer interparticle interactions with small modifications in the Monte Carlo procedure.

## Data Availability

The programs that generate the structure factors and the structure factor table in binary form are available at https://github.com/yoshi-CERMAV/hard_disk_rdf.
